# Chronic care treatment for smoking cessation in patients with serious mental illness: a pilot randomized trial

**DOI:** 10.1186/s12888-021-03113-5

**Published:** 2021-02-17

**Authors:** Andrew M. Busch, Dawn M. Nederhoff, Shira I. Dunsiger, Sandra J. Japuntich, Michelle Chrastek, Melissa Adkins-Hempel, Linda M. Rinehart, Harry Lando

**Affiliations:** 1 Department of Medicine, Hennepin Healthcare, 701 Park Avenue, S9-309, Minneapolis, MN 55415 USA; 2 Department of Medicine, University of Minnesota, 420 Delaware St. SE, Minneapolis, MN 55455 USA; 3 Division of Epidemiology and Community Health, University of Minnesota, 1300 South 2nd Street, Minneapolis, MN 55454 USA; 4 Department of Behavioral and Social Sciences, Center for Health Promotion and Health Equity, Brown University, School of Public Health, Box G-S121-8, Providence, RI 02912 USA; 5 Hennepin Healthcare Research Institute, 701 Park Avenue, PP7.700, Minneapolis, MN 55415 USA; 6 Department of Psychiatry & Behavioral Sciences, University of Minnesota, 2450 Riverside Avenue, Minneapolis, MN 55454 USA

**Keywords:** Serious mental illness, Smoking, Tobacco

## Abstract

**Background:**

Rates of smoking among those with serious mental illness (SMI) are two to three times higher than for the general population. Smoking is rarely addressed in mental health settings. Innovative outreach and treatment strategies are needed to address these disparities. The current study is a pilot study of the feasibility and acceptability of a chronic care model of tobacco cessation treatment implemented in outpatient psychiatry clinics.

**Methods:**

Participants were recruited from two outpatient psychiatric clinics and randomly assigned to intervention (counseling and nicotine replacement for 8 weeks, plus ongoing proactive outreach calls inviting reengagement in treatment) or control (brief education and referral to the state quit line). Assessments were conducted at 8 weeks (end of initial treatment block) and 6 months (end of window for retreatment). Feasibility was assessed by enrollment rate, treatment engagement, and completion of follow-up assessments. Acceptability was assessed both quantitatively and qualitatively. Preliminary efficacy was assessed by 7-day and 30-day abstinence rates, rate of quit attempts, and cigarettes per day. Psychological health was measured to assess for changes related to treatment group or attempts to quit smoking.

**Results:**

Nineteen participants were randomized to intervention and 19 to control. Recruitment proved feasible, and high rates of treatment engagement (mean of 4.5 sessions completed in initial treatment block, 89.5% uptake of nicotine replacement) and retention (94.7% of follow-up assessments completed) were observed. Treatment acceptability was high. As anticipated, there were no significant differences in abstinence between groups, but results generally favored the intervention group, including bio-verified 7-day abstinence rates of 21.1% in intervention vs. 17.6% in control and self-reported 30-day abstinence rates of 16.1% in intervention vs. 5.1% in control at 8 weeks. Significantly more intervention participants made at least one quit attempt (94.7% vs 52.6%; OR = 16.20, 95% CI: 1.79–147.01). Cigarettes per day decreased significantly more in the intervention group at 8 weeks (b = − 13.19, SE = 4.88, *p* = .02).

**Conclusions:**

It was feasible to recruit and retain SMI patients in a smoking cessation trial in the context of outpatient psychiatry. The novel chronic care model treatment was acceptable to patients and showed promise for efficacy. If efficacious, a chronic care model could be effective at reducing smoking among SMI patients.

**Trial registration:**

ClinicalTrial.gov #: NCT03822416 (registered January 30th 2019).

## Background

There has been a dramatic decrease in overall smoking prevalence in the United States [1]. However, this decline has been uneven, and there are a number of subpopulations that continue to have high rates of smoking including those with serious mental illness (SMI) [2]. In fact, rates of smoking among those with SMI are two to three times higher than those for the general US population [3]. To significantly reduce the current prevalence of tobacco use, it is essential that we reach priority populations, including those with SMI.

Life expectancy among people living with SMI is up to 20 years shorter than for the general population and tobacco use is a leading contributor to this early mortality [2, 4, 5]. Clinical practice guidelines for tobacco dependence treatment state that all smokers with psychiatric disorders should be offered treatment [6]. The American Psychiatric Association urges all mental health providers to systematically assess for smoking, recommend cessation, and provide or link to treatment [7]. Unfortunately, for a complex set of reasons, these recommendations are rarely followed in practice. Historically, smoking has been seen as normative in SMI treatment settings, especially on inpatient and residential units [8–10] and many staff in mental health settings are smokers themselves [11]. Further, some providers discourage quitting due to concern that it could exacerbate psychiatric symptoms or interfere with mental illness treatment [12, 13]. Finally, there are practical issues that limit attention given to smoking cessation in psychiatric settings, including low reimbursement rates for smoking cessation care, lack of staff trained to implement empirically supported treatment, and care systems whose resources are overwhelmed by caring for SMI symptoms and immediate basic needs [8–10, 14].

There is strong evidence, however, that SMI patients want to quit smoking and are receptive to smoking cessation treatment [15–18], that quitting does not adversely affect mental health functioning [19–22], and that smoking cessation treatments can be successful for individuals with SMI when implementation barriers can be overcome [2, 9, 23–25]. A recent clinical trial showed that proactive phone outreach to psychiatric patients to offer counseling and medication primarily through telehealth can overcome barriers to care and is effective across diagnoses [26]. A secondary analysis of this trial indicates that the proactive outreach condition was effective for patients with SMI [27]. This strategy is used in the current study.

Tobacco dependence is a chronic relapsing condition that often requires repeated intervention and multiple attempts to quit, especially among patients with SMI. However, historically most smoking cessation treatments are tested in a discrete interval of care of 3 months or less [6]. In recognition of this, experts have argued that tobacco dependence should be seen as a chronic disease that requires long-term treatment, monitoring, and retreatment following relapse [28, 29]. Alternative models consistent with a chronic disease care approach have shown promise in the general population [30–32] as well as among psychiatric patients when initiated during an inpatient psychiatric hospital stay [22].

The current study tests a novel intervention for smoking cessation in SMI patients in outpatient psychiatry settings that both utilizes proactive outreach and treats tobacco as a chronic disease that necessitates long-term monitoring and retreatment. This novel treatment avoids some of the common logistical barriers to providing evidence-based smoking cessation care to people with SMI by operating alongside, rather than within, the psychiatric treatment system (e.g., individual psychiatric providers do not need to be trained to provide cessation care; providers who still discourage or do not have time for smoking cessation can be circumvented; regular outreach attempts over time are important in recognizing tobacco dependence as a chronic disease, but psychiatric care visits are often not this frequent). We report on results of an initial randomized pilot study of this intervention with a focus on acceptability, feasibility, and preliminary efficacy.

## Methods

Participants were patients recruited from outpatient psychiatric clinics at Hennepin Healthcare (a safety net hospital system in Minneapolis, MN) and the University of Minnesota Medical School. Inclusion criteria were: 1) aged 18–65, 2) a diagnosis of a serious mental illness documented in the medical chart (i.e., schizophrenia, schizophreniform disorder, schizoaffective disorder, delusional disorder, bipolar disorder, or recurrent major depressive disorder of at least moderate severity), 3) smoking at least one cigarette daily, and 4) willing to use nicotine replacement to make a quit attempt or reduce smoking with a long-term goal of quitting. Exclusion criteria were: 1) psychiatric hospitalization within the past 6 months, 2) suicide attempt in last 6 months, 3) inability to provide valid informed consent (e.g., active psychotic episode, legal guardianship indicated in medical chart, or patient failed capacity to consent test), 4) contraindication for use of both nicotine patch and nicotine lozenge, 5) regular use of tobacco products other than cigarettes, 6) terminal illness, 7) lack of local address or telephone access, and 8) taking Clozapine or Varenicline. Those taking Clozapine were excluded because tobacco smoke effects the plasma levels of Clozapine and cessation in this context should be monitored closely by a psychiatric provider. As it is a safety issue, we confirmed self-reported Clozapine status in the medical record before enrolling. Those taking Varenicline were excluded because, at the time of the study, it was not within current standard of care to add nicotine replacement to Varenicline.

### Sample size and power

The primary aims of this study were to assess the feasibility and acceptability of the study design and the treatment manual. We originally planned to recruit up to 60 participants. Limitations on the amount and timing of funding resulted in a sample of 38. Even with the originally planned sample, however, we would not have had sufficient power to detect even large effects on the dichotomous variable of smoking cessation. We present all outcome analyses as preliminary, underpowered findings.

### Study Procedures

#### Recruitment and enrollment

The University of Minnesota (UMN) and the Hennepin Healthcare Research Institute (HHRI) institutional review boards approved all procedures. Medical chart queries identified lists of potentially eligible patients (i.e., medical chart indicated qualifying SMI diagnosis and current smoking). We briefly experimented with calling UMN patients whose smoking status was unclear in the medical chart, but this yielded a very low rate of enrollment, so it was discontinued. Patients on UMN lists had already opted in to being contacted for psychiatry clinic research. Patients on the HHRI list were mailed a letter signed by psychiatry clinic leadership informing them of the study and giving the patient the opportunity to opt out of being contacted by phone for this research.

Research staff called potentially eligible patients to introduce the study and screen those interested. Thus, recruitment was conducted using proactive outreach only (i.e., we did not do any community recruitment or accept self or provider referrals). UMN patients were recruited by phone from March to November 2019. HHRI patients were recruited by phone from July to November 2019. Those who screened eligible over the phone and continued to be interested in participating were scheduled for an in-person baseline visit at a UMN clinical research center.

Informed consent was provided in person at the beginning of this visit. As part of informed consent, participants were required to demonstrate understanding of study procedures and capacity to consent [33]. Once patients consented, they completed the baseline questionnaire and the in-person components of their assigned treatment condition during the same visit.

#### Randomization

The randomization scheme was developed in R [34] and based on a permuted block randomization procedure with small, random-sized blocks. The study statistician provided the randomization sequence and a staff member (otherwise uninvolved in randomization) created a series of numbered and ordered randomization envelopes. Randomization was not stratified and had an allocation ratio of 1:1. The envelope was only opened by the study counselor immediately before the initiation of treatment.

#### Control group

Participants randomized to the control group received a single, 20-min in-person meeting with the study counselor. During this meeting the participant received: 1) basic didactic education about the intersection of smoking and mental health, 2) a copy of the National Cancer Institute’s self-help booklet “Clearing the Air”, which the study counselor briefly reviewed with the participant, and 3) information and a brochure regarding free phone counseling and nicotine replacement available through the publicly available Minnesota quit line.

#### Intervention group

**Fig. 1:**
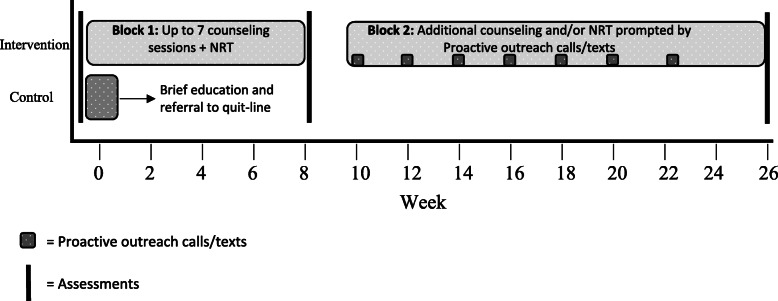
Assessments and treatment components over time

The intervention was designed to: 1) be conducted with fidelity by bachelor’s or master’s level providers who have completed Tobacco Treatment Specialist training, 2) be delivered almost exclusively by phone, 3) allow both cessation and reduction as short-term treatment goals, 4) offer only over the counter nicotine replacement for medication products (this allows for greater potential for dissemination and fewer exclusion criteria), 5) offer flexibility in the timing and number of sessions, and 6) provide long-term support for maintaining progress and retreatment as needed (consistent with a chronic care model of tobacco treatment). A graphic summary of the intervention is provided in Fig. 1.

Our manual was divided into two treatment blocks. Block 1 was acute treatment and lasted 8 weeks following randomization. Block 2 lasted from 10 to 26 weeks (i.e., 6 months) post randomization. In Block 1, participants received one 60-min, in-person counseling session immediately following randomization and up to six additional 20-min counseling sessions by phone over an eight-week period.

Block 1: During Block 1 Session 1, the counselor 1) provided education regarding the interaction of smoking and mental illness, 2) gave a strong, personalized recommendation to the participant to quit, 3) explored participants’ readiness to quit and set a specific goal for cessation or reduction, 4) reviewed past quit/reduction attempts, 5) reviewed personal reasons to quit/reduce, 6) discussed triggers to smoke and recommended strategies for management of triggers, 7) discussed obtaining social support to quit/reduce, 8) discussed craving management, 9) recommended discarding cigarettes and paraphernalia, and 10) provided options for free nicotine replacement therapy.

If planning to quit and smoking 5 or more cigarettes per day, participants were offered a choice of the nicotine patch, the nicotine lozenge, or both. Regarding the patch, those smoking > 10 cigarettes per day started on the 21 mg patch and those smoking 5–10 cigarettes per day started on the 14 mg patch. These participants could choose to use the 2 mg nicotine lozenge in addition to the nicotine patch. Participants who preferred to attempt to quit using the nicotine lozenge alone or who smoked fewer than 5 cigarettes per day were provided the 4 mg lozenge if they smoked within 30 min of awakening or the 2 mg lozenge if they smoked more than 30 min after awakening. Those participants who initially set reduction goals were offered the nicotine lozenge following the same dosing guidelines above.

At the end of Block 1 Session 1 the participants were given 4 weeks of nicotine replacement therapy. The remaining dosage of NRT was made available to the participant 4 weeks after session 1 if they were actively engaged in quitting/reduction and using nicotine replacement without side effects. Dosing, duration, and titration followed manufacturer instructions and clinical guidelines.

Block 1 Sessions 2–7 were completed by phone and included the following components: 1) assess smoking status since last session and any movement towards smoking related goals, 2) assess nicotine replacement use and side effects since last session and encourage proper nicotine replacement use, 3) assess any interaction of smoking and psychiatric symptoms or stress level, 4) collaboratively agree to a plan for changes in smoking moving forward and problem-solve barriers to that plan (concepts introduced in Session 1 were reviewed as needed). In the final call of Block 1, participants were reminded that they had the option to reengage in treatment at the upcoming Block 2 outreach contacts.

Block 2: Block 2 of the intervention started at week 10, when the counselor initiated proactive outreach phone calls or texts, which were repeated every other week through week 22. Participants chose either to receive all outreach contacts by phone or half by phone and half by text message. Outreach calls/texts were ended at week 22. This allowed at least 4 weeks remaining in the treatment window at the time of re-engagement. These calls/texts were framed as invitations to return to treatment for more support if interested. Participants who were not smoking could engage in Block 2 treatment as additional support to stay quit. Those currently smoking had to be willing to make a quit attempt or reduce smoking with a long-term goal of quitting to engage in Block 2 treatment sessions.

Interested participants who met the criteria above at any time in Block 2 were offered the chance to reengage in treatment for up to 8 weeks (or however much time remained in their Block 2 window if less than 8 weeks). Participants were encouraged to reengage in both counseling and NRT, but were allowed to reengage in either alone if they preferred.

Block 2 Session 1 was 30 min, conducted by phone, and was meant to reengage the participant in care and provide a review of concepts. Specifically, Block 2 Session 1 focused on 1) review of smoking status since last contact, 2) exploring participant’s current readiness to quit and set specific goal for cessation or reduction, 3) reviewing of reasons to quit, trigger management, craving management, obtaining social support, and removal of cigarettes/paraphernalia, and 4) options for free nicotine replacement therapy. The same protocol for nicotine replacement provision described in Block 1 above was also used for Block 2. Subsequent phone sessions in Block 2 followed the same format as Block 1 phone sessions (i.e., six additional 20 min calls were offered over 8 weeks with same length and content).

#### Retention

Participants were incentivized to complete assessments, $30 for baseline, $50 for 8 weeks, and $50 for 6 months. Transportation costs to in person visits were also reimbursed. Research staff provided multiple reminder calls before appointments and scheduling was flexible, including evening and weekend options.

#### Counselor training and treatment Fidelity

All counseling sessions were conducted by a master’s level health educator with experience conducting health behavior change coaching. The counselor completed Tobacco Treatment Specialist Training and directed readings immediately before study initiation. A licensed clinical psychologist provided 10 h of training in this treatment manual. This training included didactic instruction on smoking and the SMI population as well as demonstration and live role-plays. Throughout the study, the counselor attended weekly supervision sessions led by two licensed clinical psychologists with expertise in smoking cessation.

The counselor completed a treatment fidelity checklist at the conclusion of each session indicating which planned components of the manual were completed. All sessions were audio recorded. A random 15% of these sessions were reviewed by a licensed clinical psychologist supervisor or an experienced master’s level tobacco treatment specialist who completed the same treatment fidelity checklist for these sessions.

### Assessments

Assessments were completed by trained study staff at baseline and 8 weeks and 6 months post-randomization. Follow up assessments were completed in-person or by phone by an assessor blind to study condition. Adverse events were tracked in compliance with the institutional review boards overseeing this protocol.

#### Sociodemographic, smoking history, and psychiatric treatment utilization

At baseline, participants self-reported sociodemographic information, smoking history (including other tobacco products used), and psychiatric treatment utilization (i.e., current use of psychiatric medications and current engagement in counseling for their mental illness). Nicotine dependence was measured using the Fagerström Test for Nicotine Dependence [35].

#### Feasibility

Study feasibility was measured: by 1) flow of participants from outreach to enrollment, 2) frequency of engagement in counseling and NRT in both blocks, and 3) percentage of follow-up assessments completed.

#### Acceptability

At the 6 month follow up, treatment acceptability was measured using the Client Satisfaction Questionnaire (CSQ-8) [36] which was administered to participants in the intervention group. The CSQ-8 has a range of 8–32 and higher scores indicate greater acceptability. Both the intervention and the control groups were interviewed using open-ended qualitative questions developed by the primary investigators about their personal experience in the study.

#### Smoking outcomes

The a priori abstinence outcome was biologically verified 7-day point prevalence abstinence (7-day PPA), defined as no smoking, not even a puff, in the last 7 days. Self–reported 7-day abstinence was planned to be biologically verified by collecting a breath sample from participants using a carbon monoxide detector (< 8 ppm was considered corroboration of abstinence) at both follow-ups. However, the majority (66.7%) of 6-month assessments occurred during a COVID-19 related stay at home order that precluded in person assessments. Thus, we were unable to biologically verify abstinence for the majority of those self-reporting abstinence at 6 months. We present self-reported 7-day PPA at both 8 weeks and 6 months, as well as biologically verified 7-day PPA at 8 weeks. As secondary smoking outcomes we report on self-reported 30-day point prevalence abstinence (30 day-PPA), self-reported quit attempts (i.e., “have you stopped smoking for 24 hours or more because you were trying to quit?”; this did not include periods not smoking due to illness or hospitalization), and change in self-reported cigarettes smoked per day.

#### Psychological health outcomes

Psychological health outcomes were measured at all three time-points. Positive and negative affect were assessed using the 10 item Positive Affect Negative Affect Scales (PANAS) [37]. Depressive symptoms were assessed using the 10 item Center for Epidemiologic Studies Depression Scale (CES-D) [38]. Mental health functioning was assessed using the Mental Component Summary Scale of the 12-Item Short Form Health Survey (SF-12) [39].

#### Analysis

As a preliminary step, variables in Table 1, were summarized by treatment condition. Between group differences were examined using t-tests, chi-squared tests, and non-parametrics as appropriate. Between group baseline differences were controlled for in outcome analyses.

**Table 1:** Baseline Characteristics by Treatment Condition, Mean (SD), Median [IQR], or %

	Intervention (*N* = 19)	Control (*N* = 19)
Diagnosis per Medical Chart		
Bipolar Depression	26.3%	31.6%
Recurrent Major Depression	57.9%	63.2%
Schizoaffective Disorder	15.8%	5.3%
Age	47.89 (11.8)	50.79 (10.0)
Gender		
Male	57.9%	36.8%
Female	31.6%	63.2%
Other	10.5%	0%
Race		
American Indian/Alaska Native	5.3%	0%
Black/African American	42.1%	15.8%
White	42.1%	52.6%
Multiracial	5.3%	31.6%
Other	5.3%	0%
Hispanic/Latino Ethnicity	0%	10.5%
Education		
Less than High School	15.8%	5.6%
High School	21.1%	16.7%
At least Associate’s Degree	63.1%	77.7%
Employment		
Full Time	5.3%	42.1%
Part Time	10.5%	15.8%
Unemployed	15.8%	5.3%
Retired	10.5%	5.3%
Student	5.3%	0%
Disability	52.6%	31.6%
Total Yearly Household Income*	14,400, [12,600]	29,844 [64,800]
Cigarettes per day	16.00 (8.96)	14.53 (7.21)
Smoking Menthol Cigarettes	57.9%	47.4%
Household Smoking Ban	63.2%	52.6%
Other Tobacco Product Use		
Cigars	5.3%	5.3%
E-cigarette/Vape	5.3%	5.3%
Cigarillos	5.3%	5.3%
Cig only	84.2%	89.5%
Pipes	0%	0%
Chewing Tobacco	0%	0%
Dissolvable Tobacco	0%	0%
FTND	4.84 (2.29)	5.32 (2.03)
Taking Psychiatric Medication	78.9%	89.5%
In Counseling	26.3%	26.3%
PANAS-10 Positive Affect	14.32 (4.41)	13.63 (4.79)
PANAS-10 Negative Affect	13.47 (5.64)	16.26 (5.63)
CESD-10	14.84 (8.00)	17.74 (7.15)
SF-12 Mental Health Composite	43.68 (27.14)	32.17 (26.22)

*p < .05 for between group difference

Using a longitudinal model implemented with Generalized Estimating Equations with robust standard errors, we assessed treatment effects on self-reported 7-day PPA. Self-reported 7-day PPA at 8 weeks and 6 months was regressed on group, time, group*time, as well as baseline cofounders. A similar model was used to assess treatment effects on 30-day PPA. A series of generalized linear models with logit links were used to assess differences in biologically verified 7-day PPA and 24-h quit attempts between treatment groups controlling for confounders. A longitudinal mixed effects model with a subject specific intercept was used to examine treatment effects on smoking rate (cigarettes/day) over 6 months, while adjusting for clustering of repeated measures within participant over time, as well as baseline confounders. Next, using a series of mixed effects models, we examined treatment effects on psychological health outcomes (positive and negative affect, depressive symptoms, mental health functioning) over time. Specifically, outcomes at 8 weeks and 6 months were regressed on baseline values of the outcome, time, group, time*group, as well as baseline confounders. Finally, we compared mean psychological health outcomes over time between those quit and not quit using a series of mixed effects models with time-varying indicator of self-reported quit status.

All analyses were conducted on the intent to treat sample, with all randomized participants included in the analysis. Models used a likelihood (quasi-likelihood) approach to estimation and thus made use of all available data without directly imputing missing outcomes. A sensitivity analysis was conducted for our primary outcomes (self-reported and bio-verified 7-day PPA) controlling for presence or absence of COVID related restrictions during the participant’s treatment period. As results did not significantly differ, we present the planned analysis (not adjusting for COVID effect).

## Results

### Participants

**Fig. 2:**
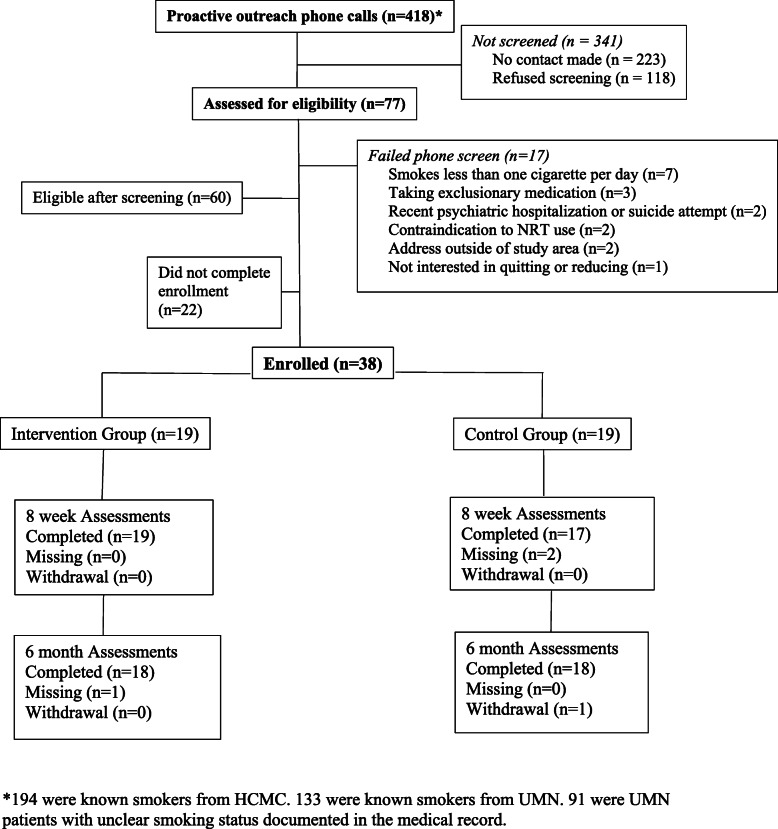
Consort diagram

Study staff attempted to contact 418 patients through proactive outreach calls. Of these calls, 327 were to patients the medical chart indicated were current smokers and 91 were to patients with an unclear smoking status in the medical record. Contact was made with 195 patients, 77 (39.5%) patients agreed to screening, 60 met eligibility criteria (77.9% of those interested), and 38 (63.3% of those qualified) consented to participate and were randomized. Nineteen participants were randomized to the intervention group and 19 to the control group. Details are reported in the consort diagram provided in Fig. 2. Qualifying diagnoses of participants were bipolar disorder (26.3% intervention, 31.6% control), recurrent major depressive disorder (57.9% intervention, 63.2% control), and schizoaffective disorder (15.8% intervention, 5.3% control). Other baseline characteristics are provided in Table 1. Treatment groups did not differ with respect to baseline characteristics with the exception of income, for which Control participants reported significantly greater household income. All outcome analyses adjusted for between condition income differences.

### Feasibility

**Fig. 3:**
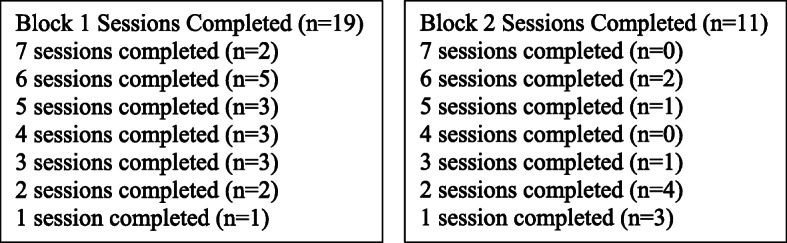
Session Completion in Each Treatment Block

9.1% of those called eventually enrolled. Among those with unclear smoking status in the chart this rate was 1.1% while among those with a clear indication of current smoking in the chart it was 11.3%. The average rate of recruitment was 4.2 participants per month. Participants in the intervention condition completed an average of 4.5 sessions in Block 1. Twelve intervention participants (63.2%) engaged in Block 2 treatment, with one engaging in NRT only, two engaging in counseling only, and nine engaging both. The 11 who engaged in Block 2 counseling completed an average of 2.8 Block 2 sessions. Figure 3 provides the distribution of session completion. 11 (57.9%) intervention participants chose an initial goal of cessation while 8 (42.1%) set an initial goal of reduction. Seventeen participants (89.5%) accepted study provided NRT, with 14 (73.7%) accepting study provided dual NRT. Use of any form of NRT at any point during the study was higher in intervention (89.5%) than in control (63.6%; *χ*^2^ = 2.91, *p* = .08).94.7% of all participants completed the 8-week and 6-month assessments.

#### Acceptability

The mean score on the CSQ for intervention participants was 27.4 (3.31), with 100% of respondents indicating they were “mostly” or “very satisfied” with the service they received. Post-treatment qualitative interviews indicated that participants appreciated the affiliation of the study with their psychiatry clinic. Specifically, they reported that this affiliation increased motivation to participate and made the offer to participate seem more “legitimate.” Those randomized to control perceived the brief education provided to be useful, but none reported significant engagement with the state quit line. Qualitative interviews also indicated that those randomized to the intervention group valued the flexibility in the treatment protocol, including when calls occurred and the option to reduce smoking as an initial goal. Intervention participants also particularly appreciated the outreach text/calls provided in Block 2, with one participant stating “No other nurse or doctor has ever followed up or helped in any way.” Taken together this indicates a high level of treatment acceptability.

#### Treatment Fidelity

On the treatment fidelity checklist, the counselor self-rated that she provided 94.0% of planned treatment components. Supervisor rated treatment fidelity checklists indicated that the counselor provided 90.4% of treatment components. Most of the missed components were due to participant factors (e.g., participant had to end call early due to limited time).

#### Self-reported 7-Day PPA

Results suggest a trend favoring the intervention condition with respect to self-reported abstinence at 8 weeks (*p* = .06). Specifically, the odds of self-reported 7-day PPA at 8 weeks was 67% higher among intervention vs. control participants (OR = 1.67, 95% CI: .98–8.53) with adjusted quit rates of 26.3% in Intervention vs. 17.6% in Control. At 6 months, there was no difference between conditions (OR = 0.39, 95% CI: .03–5.21) with adjusted 7-day PPA rates of 22.0% in intervention vs. 27.8% in control.

#### Biologically verified 7-day PPA

The odds of biologically verified 7-day PPA at 8 weeks was 19% higher among intervention vs. control participants (OR = 1.19, 95% CI: .73–8.58) with adjusted quit rates of 21.1% in intervention vs. 17.6% in control. This difference was not significant.

#### Self-reported 30-Day PPA

The odds of 30-day PPA for intervention participants was 3.38 times that of control participants at 8 weeks (OR = 3.38, 95% CI: .32–35.79; adjusted 30-day PPA rates of 16.1% in intervention vs. 5.1% in control). A similar pattern of results was seen at 6 months, where the odds of 30-day PPA for intervention participants was 2.15 times the odds for control participants (OR = 2.15, 95% CI: .21–32.39; adjusted 30-day PPA rates of 21.0% in intervention vs. 11.0% in control). There were no statistically significant differences in 30-day PPA at either timepoint.

#### 24-h quit attempts

The odds of reporting at least one 24-h quit attempt between baseline and 8 weeks for intervention participants was 2.79 times that of control participants (OR = 2.79, 95% CI: 1.06–11.10, *p* = .01; adjusted quit attempt rates of 68.4% in intervention vs. 44.0% in control). 94.7% of intervention participants reported at least one 24-h quit attempt between baseline and 6 months vs. 52.6% in control (OR = 16.20, 95% CI: 1.79–147.01, *p* = .003). Differences were statistically significant for both intervals.

#### Smoking rate

**Fig. 4:**
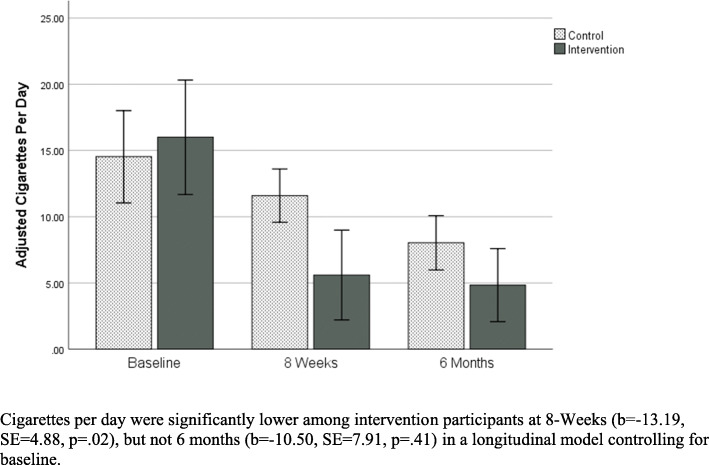
Adjusted Cigarettes per day over time

A significant effect of treatment group on smoking rate over time was observed. Specifically, mean smoking rate at 8 weeks was significantly lower among intervention participants vs. control after adjusting for baseline (b = − 13.19, SE = 4.88, *p* = .02). Although not significant, effect was in the same direction at 6 months (b = − 10.50, SE = 7.91, *p* = .41). See Fig. 4 below.

#### Psychological health outcomes

Unadjusted psychological health outcomes over time by group are presented in Table 2. Longitudinal models indicated no significant between group differences over time in positive affect (*p* = .59), negative affect (*p* = .18), depressive symptoms (*p* = .29), or mental health functioning (*p* = .43). Furthermore, there were no significant within-group changes over time in any of these measures (p’s > .10). There were no significant differences in psychological health outcomes between quitters and non-quitters over time (p’s > .10).

**Table 2:** Unadjusted Psychological Health Outcomes Across Timepoints, Mean (SD)

	Baseline Assessment	8 Week Assessment	6 Month Assessment
PANAS: Positive			
Control	13.63 (4.79)	13.31 (5.31)	13.88 (3.84)
Intervention	14.32 (4.41)	14.47 (4.30)	13.56 (4.56)
PANAS: Negative			
Control	16.26 (5.63)	15.31 (5.76)	14.63 (6.30)
Intervention	13.47 (5.64)	12.00 (4.28)	12.13 (4.01)
CES-D			
Control	17.74 (7.15)	17.06 (7.78)	15.41 (7.05)
Intervention	14.84 (8.00)	13.26 (5.76)	13.47 (5.84)
SF-12 Mental Health			
Control	32.17 (26.22)	33.44 (25.88)	37.42 (24.70)
Intervention	43.68 (27.14)	41.71 (26.63)	46.70 (16.66)

Note: Longitudinal models of psychological health outcomes indicate a) no significant between group differences over time and b) no significant within-group changes over time

#### Adverse events

No serious adverse events were reported in either group. One non-serious adverse event (increased coughing following smoking cessation) was reported in the intervention group.

## Discussion

Overall, the current study demonstrated the general feasibility of a novel, chronic care-based treatment for smoking cessation among patients with SMI and suggested areas that need attention to improve operations of a larger follow-up trial. Acceptability of treatment was high, and qualitative feedback will guide additional improvements. Preliminary smoking outcomes were promising.

Regarding feasibility of procedures, about 40% of those contacted were interested in engaging in treatment and completed a screener. This is consistent with prior work indicating that many SMI patients are open to smoking cessation treatment when offered [15–18]. Given that motivation to engage in smoking cessation treatment is variable over time, a second proactive study invitation may have yielded more patients. Almost 80% of interested patients qualified, demonstrating the feasibility of the current inclusion criteria. A future trial with greater resources can further broaden inclusion criteria (e.g., patients using Clozapine can be enrolled with a study psychiatrist to monitor them). However, fewer than two-thirds of qualifiers were randomized. This was primarily due to the logistics of scheduling a face-to-face meeting that the potential participant and a single enrollment staff member could attend. A future trial will more explicitly arrange travel and offer more flexibility regarding timing and format (including phone and/or video chat) of enrollment contacts. Assessment data were obtained from over 90% of participants at both follow-up assessment timepoints, demonstrating excellent retention feasibility and comparing favorably to retention rates of similar trials of smoking cessation among individuals with mental illness [22, 26]. COVID-19 restrictions interfered with bio-verification of abstinence at the 6-month follow-up. Methods of verification that do not require a face-to-face assessment will be explored as back-ups in future trials.

Regarding feasibility of treatment, a significant dose of counseling was delivered in Block 1 and the majority of patients accepted additional care in Block 2. However, some patients attended very few sessions. We will explore methods to overcome barriers to session engagement in future trials, including providing phone access when needed. The vast majority of patients accepted study NRT, with most accepting dual NRT. Treatment fidelity was high. Together this demonstrates that the intervention was delivered as intended and in particular that this sample of patients with SMI were interested and willing to engage in long term smoking cessation treatment. Participants also rated the intervention as highly acceptable and qualitative feedback regarding the importance of affiliation with clinical partner clinics and the perceived usefulness and uniqueness of the Block 2 outreach calls/texts will be incorporated into future trials.

All outcome data should be considered preliminary; however, some promising outcomes were observed. 7-day PPA rates were somewhat higher in the intervention condition at 8 weeks, but not at 6 months. 30-day PPA rate differences favored the intervention condition with meaningful odds ratios (2.15–3.38) but were not statistically significant at either timepoint. Those in the intervention group had a significantly higher rate of 24-h quit attempts than those in control. Together these results suggest that the intervention has promise for both increasing the rate and long-term success of quit attempts in SMI patients.

In addition, there was a significant difference in reduction in cigarettes per day favoring the intervention group at 8 weeks; this reduction was not significantly different at the 6-month follow up but was in the same direction. Intervention participants decreased smoking by about 10 cigarettes per day from baseline to 8 weeks and maintained this reduction at 6 months. There is still debate regarding the harm reduction achieved by reducing smoking [40], but the magnitude of reduction in this study suggests significant harm reduction may have been achieved. It should be noted that participants were not required to commit to a quit attempt upon enrollment, but rather could choose to reduce smoking with a future goal of quitting, and over 40% of participants chose this reduction option. Results regarding psychological health outcomes are consistent with other work indicating that smoking cessation and reduction do not worsen mental health [19, 20].

A low income and ethnically diverse sample was enrolled. This reflects the intersectionality in that recruiting SMI patients will overlap with other groups with tobacco related health disparities. However, it is notable that this sample had relatively few patients with psychotic disorders (10.5% of enrolled sample). This may reflect lower interest in quitting among those with psychotic disorders or could indicate that more support is needed to guide those with psychotic disorders through the enrollment process.

## Conclusions

Limitations include the small sample size and the lack of biochemical verification of smoking status at all timepoints due to COVID-19. We also used a single counselor for this pilot study. A future larger trial could assess possible differences in effectiveness between counselors. Further, this design does not make it clear if NRT, counseling, or both are driving between group differences. Our purpose was to test the feasibility of the chronic care model which includes both counseling and NRT in an SMI patient population. The findings were encouraging and indicate that proactive outreach and delivering counseling and NRT consistent with the chronic care model is feasible, as well as acceptable to patients with SMI. Preliminary efficacy results also were promising. A fully powered clinical trial of this treatment is warranted.

## Data Availability

Participants in this study were drawn from a small population (i.e., active patients in two psychiatry clinics) which limits our confidence that publishing the raw data could be accomplished without compromising the confidentiality of participants. Thus, we are unable to make the dataset publicly available. More detailed summary data is available from the first author to qualified investigators for appropriate purposes (e.g., conducting a meta-analysis).
